# Exploring the Dietary Behaviour of Students Who Limit Their Intake of Animal‐Source Foods: Secondary Analysis of a Nationally Representative Undergraduate Student Survey

**DOI:** 10.1111/jhn.70246

**Published:** 2026-04-14

**Authors:** Oyinlola Oyebode, Daniel Mensah

**Affiliations:** ^1^ Wolfson Institute of Population Health Queen Mary University of London London UK; ^2^ Warwick Medical School University of Warwick Coventry UK

**Keywords:** fruit and vegetable consumption, meat consumption, plant‐based diets, vegan, vegetarian

## Abstract

**Introduction:**

Plant‐based nutrition is gaining in popularity for many reasons relating to health, sustainability and animal welfare. While there is potential for diets with low or no animal source foods (ASF) to be healthy, this is not necessarily the case. Emerging adults may be at the forefront of a social movement away from ASF in the UK. This age group also have generally poor diets. Modern day plant‐based diets, particularly those followed by younger people, may include more convenience foods and less fruit and vegetables than traditional plant‐based diets. This study aims to examine whether young people following plant‐based diets consume higher or lower quality diets than young people who consume ASF (omnivores).

**Methods:**

We conducted secondary analysis of a cross‐sectional online survey of a nationally representative sample of UK university students, completed in February 2022, to explore some indicators of diet quality in students following plant‐based diets compared with omnivores. We conducted univariable and multivariable analyses (adjusted for age group, gender, ethnicity, income and parental socio‐economic group) to examine the associations between specific diets and diet indicators (fruit and vegetable intake, frequency of consumption of specific foods high in fat, sugar and/or salt, and frequency of cooking from scratch).

**Results:**

Of 2921 student participants, 1468 (50.3%) reported themselves as omnivores, 511 (17.5%) mainly vegetarian, 208 (7.1%) pescatarian, 290 (9.9%) vegetarian, and 149 (5.1%) vegan. The remainder indicated another dietary pattern or did not respond. Demographics and socio‐economic status varied significantly across diet groups. In fully adjusted models, all plant‐based diets had higher fruit and vegetable consumption than omnivores, up to 1.41 (95% CI 1.04–1.78) additional portions of fruit in pescatarians and 0.92 (0.50–1.34) additional portions of vegetables in vegans. Vegetarians and ‘mainly vegetarians' had higher odds of frequently cooking from scratch than omnivores. ‘Mainly vegetarians' were less likely than omnivores to frequently consume processed meat. Frequent consumption of other foods high in fat, sugar and/or salt was not significantly different in any of the plant‐based diets compared with omnivore diets.

**Conclusion:**

Students following plant‐based diets have higher quality diets by some indices than omnivore students.

## Key points

- Students who reported that they are “mainly vegetarian”, pescatarian, vegetarian or vegan reported higher consumption of fruit and vegetables than those who eat animal products, 2+ additional portions daily for pescatarians and vegans.
- Students who reported that they are “mainly vegetarian” or vegetarian were more likely to cook from scratch than those who eat animal products
- Although “mainly vegetarians” reported lower frequency of consumption of processed meat, there were no other differences in consumption of processed foods high in fat, salt and sugar across dietary groups.

## Graphical abstract

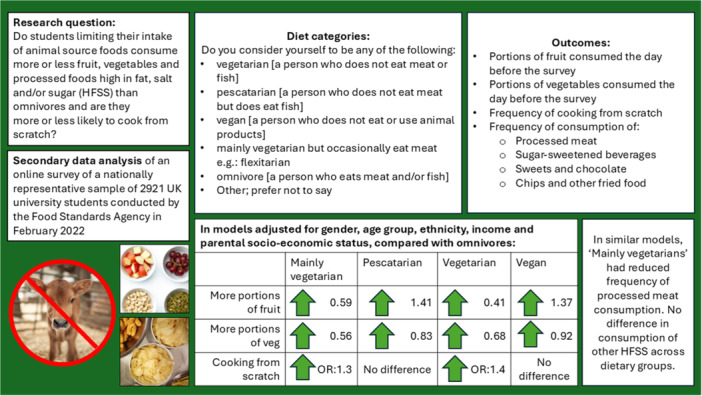

## 1 Introduction

Plant‐based diets in which the intake of animal source food (ASF) is restricted are gaining in popularity for many reasons relating to sustainability and animal welfare as well as health [1]. ASF includes meat, as well as other foods produced by animals or derived from them such as eggs, dairy products, gelatine and honey. The benefit of reducing ASF in the diet for planetary health is well established [2, 3], however the impact on health is more uncertain [2, 4].

An umbrella review of published meta‐analyses found that vegetarian diets (in which no meat is eaten) are associated with beneficial effects on blood lipid profile, diabetes, ischaemic heart disease and cancer risk [5]. An umbrella review examining the impact of a vegan diet (in which no ASF are eaten) on health found that a vegan diet was effective for reducing body weight and was associated with lower risk of cancer and potentially lower all‐cause mortality, although there was an adverse association between vegan diet and risk of fractures. For people with diabetes or at high CVD risk, a vegan diet reduced measures of adiposity, total cholesterol, LDL and improved glycaemic control [6].

Diet quality is assumed to be the mediator of any health benefits from following a plant‐based diet. A systematic review synthesising 12 studies published up to 2017 found that vegetarian diets are higher in overall diet quality compared with non‐vegetarian diets [7]. However, there are concerns that modern‐day plant‐based diets may include more convenience and highly processed foods, where traditional plant‐based diets were high in fruit, vegetables, wholegrains and pulses, and that this may reduce the potential health benefits [8, 9]. In a French prospective observational cohort, vegetarians and vegans had higher ultra‐processed food consumption than omnivores, with those who were younger or who had more recently adopted a plant‐based diet most likely to consume ultra‐processed food [10].

Emerging adults may be at the forefront of a social movement away from ASF in the UK. In the UK, this age group also have generally poor diets [11]. Students report various constraints that limit their ability to prepare and consume healthy meals including lack of skills, access, money and time [12, 13]. Compared with older adults, emerging adults are less likely to report confidence in cooking [14]. The limited data on UK student diets is mixed: students may have high intakes of confectionary and fast food alongside low consumption of food known to promote health, such as fruit and vegetables. However, there may also be substantial proportions of students who eat more healthily [15, 16, 17].

This study explores some indicators of diet quality in university students who restrict their ASF intake compared with omnivores. Specifically, we examine whether students who restrict their ASF intake consume more or less fruit, vegetables and selected ultra‐processed foods high in fat, salt and/or sugar than omnivores and whether they are more or less likely to cook from scratch.

## 2 Materials and Methods

This was secondary analysis of a cross‐sectional survey of a nationally representative sample of UK university students. The survey was co‐designed with students in 2021 and was conducted by the Food Standards Agency in February 2022. The survey's primary aim was to explore the knowledge, attitudes and behaviours of university students, as well as the experiences and challenges, relating to food safety and food insecurity, and how these vary between different groups of students. In order to ensure a representative sample from across the UK, quotas were set based on Higher Education Statistics Agency data by gender, ethnicity, region and parental socio‐economic group. A minimum of 500 students were targeted in Scotland to allow sub‐group analysis. Participants were recruited from the YouthSight panel, the largest panel of young adults in the UK, and received a £20 shopping voucher as compensation for completing the survey which was conducted online. Further details are reported on the Food Standards Agency website [18].

Within the survey, students were asked whether they follow a specific diet. They were asked “do you consider yourself to be any of the following?” and required to select one answer only. More information about each option was displayed if they clicked on an info button. The options were: vegetarian [a person who does not eat meat or fish]; pescatarian [a person who does not eat meat but does eat fish]; vegan [a person who does not eat or use animal products]; mainly vegetarian but occasionally eat meat e.g.: flexitarian; omnivore [a person who eats meat and/or fish]; other; prefer not to say. Those who indicated “other” were able to describe in an open text box.

Students were asked about their fruit and vegetable consumption the day before the survey. They were asked:

1. Thinking about what you ate and drank yesterday. How many portions of vegetables did you eat yesterday? A portion is around 3 heaped tablespoons of beans or pulses or cooked vegetables such as carrots or peas, a handful of cherry tomatoes or a bowl of salad. Please include salad, fresh, frozen or tinned vegetables but do not include potatoes.
2. Thinking about what you ate and drank yesterday. How many portions of fruit did you eat yesterday? A portion is one slice of large fruit such as a pineapple or melon, an apple, banana or pear, two small pieces of fruit such as satsumas or plums, a handful of grapes or 1 tablespoon of dried fruit. Please include fresh, frozen, tinned or dried fruit. Do not include fruit juice.

Students were also asked how often they ate a range of processed foods high in fat, salt or sugar. Specifically they were asked: how often do you eat: processed meat products (e.g.: sausages, burgers, meat and chicken pies); sweets and chocolate; sugary fizzy drinks and diluted squash; chips and other fried foods and asked to select one answer for each statement from the following options: Every day; Most days; 2–3 times a week; About once a week; 2–3 times a month; About once a month; Less than once a month; Never; Can't remember. Students who had indicated that they followed pescatarian, vegetarian or vegan diets were not asked about processed meat products. We re‐coded the responses to this question into a binary variable: those who consumed the selected processed foods 2–3 times a week or more vs those who consumed them less frequently. Those who indicated that they “can't remember” were excluded from these analyses.

Students were asked “How often do you cook a meal from scratch/from basic ingredients at your term‐time residence?” For example, using unprocessed fruit, vegetables, eggs, meat or rice without the use of readymade sauces, spice mixes or processed meat such as chicken nuggets or burgers. They were asked to select one answer from the following options: at least daily; 2–3 times a week; once a week; less than once a week but more than once a month; once a month; less often than once a month, but more than four times a year; less than 4 times a year; never; don't know. We re‐coded the responses to this question into a binary variable: those who cooked from scratch 2–3 times a week or more vs those who cooked from scratch less frequently. Those who indicated “don't know” were excluded from these analyses.

Students were also asked to report their socio‐demographic characteristics including age, gender, ethnicity, annual income (including their student loan, income from working, grants and any money from parents, relatives or guardians), and parental socio‐economic group (occupation of the chief income earner in your parental/guardian household).

We examined the characteristics of participants by declared diet, testing for differences between groups uses chi^2^. We conducted linear regression analyses to examine the associations between declared diets and consumption of fruit and vegetable portions, and logistic regression analyses to test the associations between declared diet and the frequent consumption of processed meat products; sweets and chocolate; sugary fizzy drinks and diluted squash; chips and other fried foods and cooking from scratch. We fitted both univariable and multivariable models in both the linear and logistic regressions. Multivariable analyses were adjusted for potential confounders available in the dataset: age group, gender, ethnicity, income and parental socio‐economic group.

## 3 Results

### 3.1 Characteristics of Participants

A final sample of 2921 students took part in the online survey of whom the majority were women (62.4%), white British (73.2%) and aged 17–21 years (74.3%) [Table 1]. Of the 2921 participants, 1468 (50.3%) reported themselves as omnivores, 511 (17.5%) as mainly vegetarian, 208 (7.1%) as pescatarian, 290 (9.9%) as vegetarian, 149 (5.1%) as vegan, 136 (4.7%) as “other” often described in the free‐text as “carnivore” “meat lover” etc or “halal” “kosher” or avoiding other food groups (mainly dairy and gluten). 159 students (5.4%) did not disclose their diet.

**Table 1:** Characteristics of participants.

	Omnivore	Mainly vegetarian, occasionally eat meat	Pescatarian	Vegetarian	Vegan	Other/Missing	Total (% reported for the column)	Chi^2^
*N* (%)	1,468 (50.3%)	511 (17.5%)	208 (7.1%)	290 (9.9%)	149 (5.1%)	295 (10.1%)	2,921 (100.0%)	
**Age group**								< 0.001
17–18	314 (52.9%)	96 (16.2%)	33 (5.6%)	53 (8.9%)	33 (5.6%)	65 (10.9%)	594 (20.3%)	
19	385 (56.8%)	101 (14.9%)	44 (6.5%)	52 (7.7%)	23 (3.4%)	73 (10.8%)	678 (23.2%)	
20	234 (47.6%)	81 (16.5%)	33 (6.7%)	69 (14.0%)	23 (4.7%)	52 (10.6%)	492 (16.8%)	
21	201 (49.1%)	86 (21.0%)	24 (5.9%)	46 (11.3%)	23 (5.6%)	29 (7.1%)	409 (14.0%)	
22	96 (41.2%)	35 (15.0%)	39 (16.7%)	22 (9.4%)	24 (10.3%)	17 (7.3%)	233 (8.0%)	
23–29	156 (47.6%)	70 (21.3%)	24 (7.3%)	23 (7.0%)	14 (4.3%)	41 (12.5%)	328 (11.2%)	
30+	82 (43.9%)	42 (22.5%)	11 (5.9%)	25 (13.4%)	9 (4.8%)	18 (9.6%)	187 (6.4%)	
**Gender**								< 0.001
Men	530 (50.8%)	160 (15.3%)	105 (10.7%)	85 (8.2%)	69 (6.6%)	94 (9.0%)	1,043 (35.7%)	
Women	915 (50.2%)	342 (18.8%)	97 (5.3%)	199 (10.9%)	76 (4.2%)	193 (10.6%)	1,822 (62.4%)	
Other	23 (41.1%)	9 (16.1%)	6 (10.7%)	6 (10.7%)	4 (7.1%)	8 (14.3%)	56 (1.9%)	
**Ethnicity**								< 0.001
Asian	157 (47.3%)	59 (17.8%)	2 (0.6%)	43 (13.0%)	11 (3.3%)	60 (18.1%)	332 (11.4%)	
Black	55 (67.1%)	7 (8.5%)	5 (6.1%)	6 (7.3%)	2 (2.4%)	7 (8.5%)	82 (2.8%)	
Mixed	64 (56.6%)	24 (21.2%)	3 (2.65%)	11 (9.7%)	1 (0.9%)	10 (8.9%)	113 (3.9%)	
White British	1057 (49.4%)	370 (17.3%)	191 (9.0%)	209 (9.8%)	132 (6.2%)	180 (8.4%)	2,139 (73.2%)	
White other	71 (52.6%)	27 (20.0%)	4 (3.0%)	14 (10.4%)	2 (1.5%)	17 (12.6%)	135 (4.6%)	
Other/missing	64 (53.3%)	24 (20.0%)	3 (2.5%)	7 (5.8%)	1 (0.8%)	21 (17.5%)	120 (4.1%)	
**Annual income**								< 0.001
Less than £13,000	766 (56.2%)	218 (16.0%)	44 (3.2%)	132 (9.7%)	51 (3.7%)	153 (11.2%)	1,364 (46.7%)	
£13,000–£18,999	181 (47.5%)	65 (17.1%)	37 (9.7%)	46 (12.1%)	21 (5.5%)	31 (8.14%	381 (13.0%)	
£19,000–£25,999	109 (43.4%)	58 (23.1%)	32 (12.8%)	25 (10.0%)	14 (5.6%)	13 (5.2%)	251 (8.6%)	
£26,000–£31,999	84 (40.4%)	40 (19.2%)	33 (15.9%)	16 (7.7%)	22 (10.6%)	13 (6.3%)	208 (7.1%)	
£32,000–£47,999	81 (40.3%)	39 (19.4%)	38 (18.9%)	12 (6.0%)	17 (8.5%)	14 (7.0%)	201 (6.9%)	
£48,000+	88 (38.6%)	55 (24.1%)	16 (7.0%)	30 (13.2%)	14 (6.1%)	25 (11.0%)	228 (7.8%)	
Other	159 (55.2%)	36 (12.5%)	8 (2.8%)	29 (10.1%)	10 (3.5%)	46 (16.0%)	288 (9.9%)	
**Parental SES**								< 0.001
Professional/higher managerial role	245 (50.3%)	89 (18.2%)	37 (7.6%)	47 (9.7%)	27 (5.5%)	42 (8.6%)	487 (16.7%)	
Manager/senior administrator	337 (43.0%)	150 (19.1%)	89 (11.4%)	88 (11.2%)	63 (8.1%)	56 (7.2%)	783 (26.8%)	
Supervisor/clerical/skilled non‐manual	320 (49.9%)	124 (19.3%)	51 (8.0%)	58 (9.1%)	31 (4.8%)	57 (8.9%)	641 (21.9%)	
Skilled manual worker	185 (57.6%)	53 (16.5%)	8 (2.5%)	23 (7.2%)	13 (4.1%)	39 (12.2%)	321 (11.0%)	
Semi‐skilled/unskilled manual worker	183 (56.7%)	43 (13.3%)	13 (4.0%)	38 (11.8%)	8 (2.5%)	38 (11.8%)	323 (11.1%)	
Receiving state benefits for sickness, unemployment, old age or any other reason	49 (54.4%)	15 (16.7%)	2 (2.2%)	7 (7.8%)	3 (3.3%)	14 (15.6%)	90 (3.1%)	
Others	149 (54.0%)	37 (13.4%)	8 (2.9%)	29 (10.5%)	4 (1.5%)	49 (17.6%)	276 (9.4%)	

All the characteristics examined were significantly associated with declared diet (*p* < 0.001 for all characteristics) [Table 1]. Younger participants were more likely to be omnivores than older participants. Women were more likely to be mainly vegetarian or vegetarian than men, while men were more likely to be omnivore, pescatarian and vegan. Those indicating another gender were least likely to be omnivore. Asian students were least likely to be omnivore while black students were most likely to be omnivore. The percentage of omnivores reduced as income increased and generally there were more omnivores among students with parents in lower socioeconomic positions.

### 3.2 Univariable Analyses

The association between the portions of fruit and vegetables consumption and declared diets is shown in Table 2. All diets in which ASF are limited had higher consumption of both fruit and vegetables than omnivores (the reference category), with pescatarians and vegans consuming more than 2 extra portions of fruit (2.35 (95%CI: 1.95–2.75) and 2.08 (1.62–2.54), respectively) daily. Pescatarians and vegans also consumed more than 1 extra portion of vegetables per day (1.45 (1.08–1.83) and 1.40 (0.96–1.83), respectively).

**Table 2:** Univariable analysis: association between fruit and vegetable portions consumed and declared diets with omnivores as the reference category.

		Declared diet				
		Mainly vegetarian	Pescatarian	Vegetarian	Vegan	Other/Missing
**Portions of Fruit**	Coefficient (95% CI)	0.81 (0.53 to 1.09)	2.35 (1.95 to 2.75)	0.48 (0.14 to 0.83)	2.08 (1.62 to 2.54)	0.41 (0.07 to 0.76)
	*p*‐value	< 0.001	< 0.001	0.006	< 0.001	0.017
**Portions of veg**	Coefficient (95% CI)	0.71 (0.45 to 0.97)	1.45 (1.08 to 1.83)	0.76 (0.43 to 1.09)	1.40 (0.96 to 1.83)	0.33 (0.00 to 0.66)
	*p*‐value	< 0.001	< 0.001	< 0.001	< 0.001	0.045

Univariable analyses of other dietary behaviour and declared diet showed mixed results [Table 3]. Those reporting themselves to be mainly vegetarian or vegetarian were more likely than omnivores to also report frequently cooking from scratch (OR 1.36 (1.10–1.67) and 1.40 (1.08–1.82) respectively). Those reporting “other” or missing data on declared diets were significant less likely to frequently cook from scratch that omnivores and no other dietary pattern was significantly different from omnivores.

**Table 3:** Univariable analysis: association between various dietary behaviours and declared diets with omnivores as the reference category.

		Declared diet				
		Mainly vegetarian	Pescatarian	Vegetarian	Vegan	Other/Missing
**Frequency of cooking from scratch**	Odds ratio (95% CI)	1.36 (1.10 to 1.67)	0.84 (0.63 to 1.12)	1.40 (1.08 to 1.82)	0.84 (0.60 to 1.17)	0.74 (0.57 to 0.95)
	*p*‐value	0.004	0.240	0.011	0.295	0.018
**Frequency of processed meat products consumption**	Odds ratio (95% CI)	0.68 (0.55 to 0.84)				1.18 (0.83 to 1.68)
	*p*‐value	< 0.001				0.358
**Frequency of fizzy/sugar sweetened beverages consumption**	Odds ratio (95% CI)	0.89 (0.72 to 1.09)	1.21 (0.90 to 1.62)	0.84 (0.65 to 1.09)	1.44 (1.03 to 2.02)	1.23 (0.96 to 1.59)
	*p*‐value	0.252	0.204	0.199	0.035	0.107
**Frequency of sweets and chocolate consumption**	Odds ratio (95% CI)	0.93 (0.76 to 1.15)	0.84 (0.62 to 1.13)	1.34 (1.02 to 1.76)	0.76 (0.54 to 1.08)	1.08 (0.83 to 1.42)
	*p*‐value	0.498	0.250	0.039	0.124	0.553
**Frequency of chips and other fried foods consumption**	Odds ratio (95% CI)	0.94 (0.76–1.16)	1.16 (0.86–1.56)	1.19 (0.92–1.54)	1.42 (1.01–2.01)	1.47 (1.14–1.90)
	*p*‐value	0.575	0.321	0.185	0.042	0.003

Those reporting themselves to be mainly vegetarian had lower frequency of consumption of processed meat products than omnivores. This was not assessed for the other diets in which ASF are limited. Vegans were more likely to frequently consumed sugar sweetened beverages than omnivores (OR 1.44 (1.03–2.02)) as well as more frequently consume chips and other fried food (1.42 (1.01–2.01). Vegetarians were more likely to report frequent consumption of sweets and chocolate than omnivores (OR 1.34 (1.02–1.76)). There were no other significant differences across declared diet groups.

### 3.3 Multivariable Analyses

In the fully adjusted models, all diets in which ASF are limited had higher consumption of both fruit and vegetables than omnivores [Table 4]. For daily fruit this ranged from 0.40 (0.09–0.72) more portions in vegetarians, 0.59 (0.34–0.84) in mainly vegetarians, 1.41 (1.04–1.78) in pescatarians and 1.37 (0.95–1.79) in vegans. For daily vegetables this ranged from 0.56 (0.31–0.81) more portions for mainly vegetarians, through 0.68 (0.37–0.99) in vegetarians, 0.83 (0.46–1.20) in pescatarians and 0.92 (0.50–1.34) in vegans.

**Table 4:** Multivariable analysis: association between fruit and vegetable portions consumed and declared diets, with omnivores as the references category. Adjusted for gender, age group, ethnicity, income and parental SES.

		Mainly vegetarian	Pescatarian	Vegetarian	Vegan	Other/Missing
**Portions of fruit consumed**	Coefficient (95% CI)	0.59 (0.34 to 0.84)	1.41 (1.04 to 1.78)	0.41 (0.09 to 0.72)	1.37 (0.95 to 1.79)	0.51 (0.20 to 0.82)
	*p*‐value	< 0.001	< 0.001	0.011	< 0.001	0.001
**Portions of veg consumed**	Coefficient (95% CI)	0.56 (0.31 to 0.81)	0.83 (0.46 to 1.20)	0.68 (0.37 to 0.99)	0.92 (0.50 to 1.34)	0.40 (0.09 to 0.71)
	*p*‐value	< 0.001	< 0.001	< 0.001	< 0.001	0.011

Multivariable analyses examining the other dietary behaviours found that those reporting themselves to be mainly vegetarian had significantly higher odds of frequently cooking from scratch (OR 1.34 (1.08–1.65)) as did vegetarians (1.37 (1.05–1.79)) [Table 5]. Those who were mainly vegetarian were also less likely than omnivores to frequently consume processed meat (0.66 (0.53–0.83)). The frequent consumption of other food high in fat, salt and sugar was not significantly different in any of the declared diets compared with omnivore diets except for those who selected “other” or did not declare their dietary pattern [Table 5].

**Table 5:** Multivariable analysis: association between various dietary behaviours and declared diets, with omnivores as the references category. Adjusted for gender, age group, ethnicity, income and parental SES.

		Mainly vegetarian	Pescatarian	Vegetarian	Vegan	Other/Missing
**Frequency of cooking from scratch**	Odds ratio (95% CI)	1.34 (1.08 to 1.65)	0.91 (0.67 to 1.24)	1.37 (1.05 to 1.79)	0.87 (0.61 to 1.23)	0.76 (0.59 to 0.98)
	*p*‐value	0.008	0.548	0.022	0.416	0.038
**Frequency of processed meat consumption**	Odds ratio (95% CI)	0.66 (0.53 to 0.83)				1.35 (0.93 to 1.95)
	*p*‐value	< 0.001				0.110
**Frequency of sugar‐sweetened beverage consumption**	Odds ratio (95% CI)	0.86 (0.70 to 1.07)	1.10 (0.81 to 1.49)	0.81 (0.62 to 1.06)	1.30 (0.91 to 1.84)	1.27 (0.98 to 1.65)
	*p*‐value	0.169	0.556	0.127	0.146	0.074
**Frequency of sweets and chocolate consumption**	Odds ratio (95% CI)	0.93 (0.75 to 1.16)	1.02 (0.74 to 1.40)	1.31 (0.98 to 1.73)	0.84 (0.58 to 1.19)	1.07 (0.81 to 1.41)
	*p*‐value	0.532	0.918	0.065	0.322	0.632
**Frequency of chips and other fried foods consumption**	Odds ratio (95% CI)	0.93 (0.75 to 1.15)	1.05 (0.77 to 1.44)	1.18 (0.91 to 1.54)	1.32 (0.93 to 1.88)	1.50 (1.16 to 1.95)
	*p*‐value	0.498	0.758	0.217	0.123	0.002

## 4 Discussion

In this representative sample of UK undergraduate students, following a diet in which the amount of ASF is restricted was associated with increased consumption of fruit and vegetables compared with omnivores. Particularly, pescatarian and vegan students may consume at least two portions of fruit and vegetables more than omnivore students daily, after adjustment for socio‐demographic characteristics. UK students who are vegetarians and mainly vegetarian are more likely to frequently cook from scratch than omnivores. Frequency of consumption of foods high in fat, salt, and/or sugar appeared to be similar across all groups of students regardless of whether they limit ASF in their diets.

Our findings are in line with the wider literature in which those who limit their intake of ASF have generally been found to have better diet quality [7]. More specifically, our findings match those from a study of 165 Norwegian adults aged 16–24, which found that vegans and flexitarians consumed vegetables, beans and lentils more frequently than omnivores with no differences in sugary food and salty food consumption across dietary groups [19]. Additionally, our finding that vegetarian and flexitarian students were more likely to cook from scratch is in line with a study of 1448 students from 5 UK universities which found that students with greater cooking ability tended towards a vegetarian eating pattern [16].

In order to meet international and national targets on climate change and planetary health, the population of the UK needs to reduce ASF consumption [20]. Although there have been concerns that those adopting plant‐based diets which limit ASF, particularly in younger age‐groups may be consuming less healthy diets than previous traditional plant‐based diets [8, 9], in our study, young adults limiting their intake of ASF are consuming more fruit and vegetables, and, for some groups, cooking from scratch more frequently, while not consuming significantly more foods high in fat, salt and/or sugar. Higher fruit and vegetable consumption is generally associated with better health [21]. Frequently cooking from scratch is also associated with higher overall diet quality, better nutrition knowledge and lower ultra‐processed food consumption [22, 23]. In a prospective cohort study being actively involved in preparing one's own food was positively associated with a reduction in the risk of obesity [24]. This suggests that encouraging or supporting adoption of plant‐based diets in this age group may have positive co‐benefits for health.

The strengths of our study are that we have examined a range of different declared diets and a number of indicators of dietary behaviour. We were able to adjust our analyses for possible confounders. Also, we used a survey that was designed to give a nationally representative sample of students from across the UK. However, differential response rates within the quotas set by the survey team means that the final sample may not be fully representative of the student body from which the sample was recruited. Higher education student statistics for 2022/23 reports that 57% of undergraduates were female, 59% were of white ethnicity and 51% were aged 20 and under, suggesting our sample was more female, white and younger than the national average [25]. We did not find data on student income and parental socioeconomic status that we could compare with our sample, but the sample may also not be fully representative on these criteria. The study also has several limitations: the outcome variables were all self‐reported and the questions used have not been previously validated, to our knowledge. We were only able to examine some indications of dietary behaviour but do not have comprehensive information on dietary consumption, so we cannot say whether the students limiting ASF have healthier diets overall, only that they do by some indicators. We are unable to disaggregate within the fruit and vegetable categories to see which fruits and vegetables in particular are being consumed‐ for example to explore whether the increased vegetable consumption seen is due to beans and pulses replacing meat for some dietary groups. It is worth noting that in the unadjusted analyses some of the diets with limited ASF were associated with increased consumption of some of the foods high in fat, salt and sugar. In addition, while meat‐eaters were asked about consumption of processed meat products, vegetarians and vegans were not asked about processed meat alternatives which could also lead to missing some equivalent high fat, salt and sugar foods in the diets of those limiting ASF. Sensitivity analysis could have included recoding some of the students who gave responses that suggested that they were omnivores with a high‐probability to the omnivore group. We didn't do this as every response was unique among the 295 students who indicated they followed an “other” diet and we thought our interpretations may not always be right (e.g., if someone listed foods they avoid and none of these were meat, we could consider that they are probably an omnivore but could not be certain). This group generally seemed similar to the omnivores, with slightly higher fruit and vegetable consumption but slightly less likely to cook from scratch and more likely to eat the foods high in fat, salt and sugar. Re‐categorisation of a small number of them probably wouldn't have made a large difference to the overall findings. Further research to examine macro and micronutrient intakes in this age group by declared diet would be needed before being able to be certain that students following diets limiting ASF are eating more healthily than omnivore students.

## 5 Conclusion

Students following plant‐based diets have higher quality diets by some indices than omnivore students.

## Author Contributions

Oyinlola Oyebode conceptualised the study, planned the methodology and completed data analysis. Oyinlola Oyebode wrote the original draft. Daniel Mensah acquired the funding and the data, reviewed and edited the manuscript.

## Funding

The authors have nothing to report.

## Ethics Statement

This manuscript reports secondary data analysis of a fully anonymised dataset collected by the Food Standards Agency, accessed with permission of the Agency. For this reason, no ethical approval was sought.

## Conflicts of Interest

The authors declare no conflicts of interest.
